# An Energy-Efficient Clustering Routing Protocol for Wireless Sensor Networks Based on AGNES with Balanced Energy Consumption Optimization

**DOI:** 10.3390/s18113938

**Published:** 2018-11-14

**Authors:** Zhidong Zhao, Kaida Xu, Guohua Hui, Liqin Hu

**Affiliations:** 1 Hangdian Smart City Research Center of Zhejiang Province, Hangzhou Dianzi University, Hangzhou 310018, China; 2 College of Electronics and Information, Hangzhou Dianzi University, Hangzhou 310018, China; 3 School of Communication Engineering, Hangzhou Dianzi University, Hangzhou 310018, China; 4 Key Laboratory of Forestry Intelligent Monitoring and Information Technology of Zhejiang Province, School of Information Engineering, Zhejiang A & F University, Linan 311300, China; guohua_hui@zafu.edu.cn; 5 Department of Construction Engineering, Zhejiang College of Construction, Hangzhou 311231, China; huliqin98@gmail.com

**Keywords:** wireless sensor network, AGglomerative NESting, dual-cluster heads, dormancy, throughput, lifetime

## Abstract

To further prolong the lifetime of wireless sensor network (WSN), researchers from various countries have proposed many clustering routing protocols. However, the total network energy consumption of most protocols is not well minimized and balanced. To alleviate this problem, this paper proposes an energy-efficient clustering routing protocol in WSNs. To begin with, this paper introduces a new network structure model and combines the original energy consumption model to construct a new method to determine the optimal number of clusters for the total energy consumption minimization. Based on the balanced energy consumption, then we optimize the AGglomerative NESting (AGNES) algorithm, including: (1) introduction of distance variance, (2) the dual-cluster heads (D-CHs) division of the energy balance strategy, and (3) the node dormancy mechanism. In addition, the CHs priority function is constructed based on the residual energy and position of the node. Finally, we simulated this protocol in homogeneous networks (the initial energy = 0.4 J, 0.6 J and 0.8 J) and heterogeneous networks (the initial energy = 0.4–0.8 J). Simulation results show that our proposed protocol can reduce the network energy consumption decay rate, prolong the network lifetime, and improve the network throughput in the above two networks.

## 1. Introduction

As a symbol of the 4th generation of sensor networks, wireless sensor network (WSN) is a distributed self-organizing network that integrates data acquisition, processing and communication functions. It has a wide range of applications in many important fields, such as agriculture, transportation, and military. Usually, the nodes are powered by limited batteries, so the purpose of extending the lifetime of WSN can be achieved by reducing the energy consumption.

**Figure 1:**
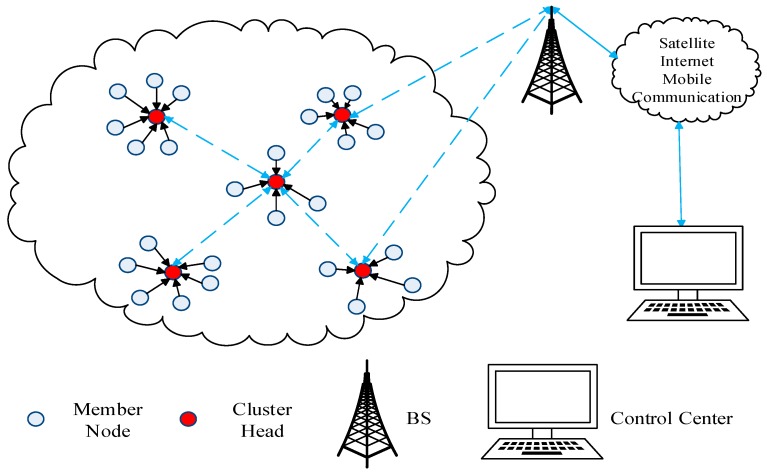
A typical wireless sensor network (WSN) logical hierarchy diagram. BS: base station.

As an effective scheme to save energy consumption of WSN, a reasonable clustering routing protocol is generally divided into three phases: cluster setup phase, cluster heads (CHs) election phase, and data transmission phase. In the cluster setup phase, the sensor node groups in the detection area form clusters of different sizes. Based on a certain electoral mechanism, some nodes are selected as the CHs and the remaining nodes act as the member nodes in the CHs election phase. Finally, in the data transmission phase, the member nodes are responsible for collecting environmental information and then transmitting it to the CHs. After the aggregation and data fusion, the CHs send it to the base station (BS). The latter transmits it to the control center (CC) via satellite, Internet, or a mobile communication network, eventually the center personnel make decisions based on current environmental information. Figure 1 shows a typical WSN logical hierarchy diagram.

In recent years, researchers in various countries have proposed various kinds of clustering protocols for WSNs. There are several classical protocols, such as LEACH (Low-Energy Adaptive Cluster Hierarchical) [1,2], SEP (Stable Election Protocol) [3], DEEC (Distributed Energy-Efficient Clustering) [4], and HEED (Hybrid Energy Efficient Distribution) [5].

A new group-based cluster-based hierarchical partitioning scheme that minimizes the number of hops in a cluster is proposed in [6] and a hybrid clustering method combining static and dynamic clustering is proposed in [7]. Elhabyan, R., W. Shi and M. St-Hilaire [8] propose multi-objective evolutionary algorithms (MOEAs) to obtain the optimal network configuration. A Chain Based Cluster Cooperative Protocol (CBCCP) is proposed in [9] and Markov model is considered in [10]. Wang, Q. et al. [11] propose a new network structure model, then according to the original energy consumption model [12], the formula for determining the optimal cluster number of WSN in the region is proposed. 

In addition, the proposed protocol introduces some common algorithms, such as ant colony optimization (ACO) [13,14], particle swarm optimization (PSO) [15,16], principal component analysis (PCA) [17], harmony search algorithm (HSA) [18,19], spectral partitioning [11] and so on. 

It is not difficult to find that the protocols have been improved in the following aspects: (1) intra-cluster data transmission path [20], (2) inter-cluster data transmission path, (3) data transmission amount compression [21], (4) implementation of mobile BS or relay node [22], (5) data security consideration [23], (6) increase in the number of sink nodes [24], (7) CHs selection mechanism optimization [21,25,26].

As the WSN works continuously, the nodes will eventually die due to the continuous loss of energy. The problems faced by energy consumption in WSN mainly include two aspects: (1) The large total energy consumption; (2) The unbalanced energy consumption. (1) will cause the average energy consumption of the nodes in the network to be too large, resulting in a decline in the overall performance of WSN. (2) will cause a large difference in the death time of the node groups in WSN, which will adversely affect the stability of the network and the efficiency of information transmission. From the perspective of efficient energy and balanced energy consumption, this paper proposes an energy-efficient clustering routing protocol in WSNs. The basic premise is as follows: To minimize the total energy consumption of WSN, we propose a new network structure model and derive the optimal number of clusters according to the former and the original energy consumption model [12]. The clusters generated by traditional clustering routing protocols tend to be of different sizes, resulting in unbalanced energy consumption (i.e., the energy consumption of CHs in large clusters is often much larger than that in small clusters.). To alleviate this problem, based on the distance, the variance is introduced to reduce the difference in the distance between the nodes within the clusters in the cluster setup phase. Then in the CHs election phase, we implement the D-CHs division of the energy balance strategy and the node dormancy mechanism for the large cluster area before and after the death of the first node, respectively. In terms of optimal CHs election, we take the position of the node into account apart from the residual energy, which is obviously different from traditional protocols considering only the residual energy.

The main innovative points of this paper include the following:

- The optimal number of clusters is derived to minimize the total energy consumption of WSN.
- Variance introduction, the D-CHs division of the energy balance strategy and the node dormancy mechanism are necessary to enable the energy consumption balance.
- A new CHs priority function can ensure the nodes with better positions and adequate residual energy could have higher probabilities to be CHs.
- The new clustering routing protocol achieves good network performance, including lifetime, energy consumption, and throughput.

As for the communication technology, the previous Bluetooth [27] has high system complexity, short transmission distance, and large power consumption, which is not popular in WSN. In contrast, ZigBee has a wide range of applications in WSN due to its simplicity, low power consumption, low cost, and long-distance transmission.

In 2016, Bluetooth 5 [28,29] came into being. Compared with the previous Bluetooth version, its maximum transmission distance is increased 3 times, the power is greatly reduced, and the transmission rate is significantly improved. In addition, it will increase the maximum data capacity to 255 bytes, while ZigBee has only 100 bytes in this aspect. Thus, Bluetooth 5 is gradually becoming a new generation of Internet of Things communication technology. In this paper, we choose Bluetooth 5 as the communication technology of WSN.

The rest of this paper is organized as follows: Section 2.1 introduces a new network structure model, Section 2.2 describes the original energy consumption model, and Section 2.3 proposes a new method for determining the optimal number of clusters. Section 3 describes the details of the protocol. The simulation study is conducted in Section 4. Finally, Section 5 summarizes the research and prospects for the future.

## 2. Network Model and Optimal Cluster Number Calculation

In this section, we propose a new network structure model and quote the energy consumption model proposed in [12], then suggest a new method to determine the optimal cluster number.

### 2.1. Network Model

The network model used in this paper is a WSN model in which *N* sensor nodes are evenly arranged in a circular area of diameter *M*. The BS in the center of the network area has strong computing power. Because the BS energy can be self-replenished, the energy loss of the BS is not considered in this work. On this basis, we can make the following assumptions about the WSN:

(1) All sensor nodes are static, nodes transmit data to each other in single or multiple hops, and the node energy cannot be supplemented.
(2) The idealized simulation environment does not consider the influence of natural factors such as temperature, humidity, light, and wind on the sensor nodes.

### 2.2. Energy Consumption Model

This paper quotes the energy consumption model proposed in [12]. According to the actual transmission distance from the CHs to the BS, the free space model and the multipath fading channel model both need to be comprehensively analyzed, which is different from [12] considering only the multipath fading channel model. Therefore, the expression of the total energy consumption of the model will undergo some changes. 

*E_T_(e*,*d*) indicates the energy consumed by the wireless transmitter to transmit a set of *e* bits of information. The expression is as follows:

(1) $$\;E_{T}\left(e,d\right)=\{\begin{array}{l} e\times \left(E_{elec}+\varepsilon_{fs}d^{2}\right),\;d<d_{0}\\ e\times \left(E_{elec}+\varepsilon_{mp}d^{4}\right),\;d\geq d_{0} \end{array}\;$$

*E_R_*(*e*) indicates the energy required to receive the information of the *e* bit. The expression is as follows:

(2) $$\;E_{R}\left(e\right)=e\times E_{elec}\;$$

In Equations (1) and (2), *E_elec_* is the energy consumed per bit by the transmitter or receiving circuit and *d* is the distance between the transmitter and the receiver. In Equation (2), when *d* < *d*_0_, we use the free space model and *ε_fs_* acts as the energy factor per bit. Otherwise, the multipath fading channel model is used, and *ε_mp_* acts as the energy factor per bit. In addition, *d*_0_ is used as the distance threshold. As long as it is input as an independent variable into the free space model and the multipath fading channel model to establish an equation, the following expression can be obtained:

(3) $$d_{0}=\sqrt{\frac{\varepsilon_{fs}}{\varepsilon_{mp}}}\;$$

In each round of data transmission, the cluster member nodes are responsible for sensing information from the environment, then transmitting it to the CH of the corresponding cluster. Therefore, the calculation formula for the energy consumed by transmitting *e* bit information is defined as follows:

(4) $$E_{non-CH}=e\cdot E_{elec}+e\cdot \varepsilon_{fs}d_{toCH}^{2}\;$$

In Equation (4), *d_toCH_* represents the distance from the cluster member node to the CH. 

The CH receives information from the cluster member nodes in the cluster, then fuses the information with that which it senses from the environment, eventually transmits the merged information to the BS. In this paper, we assume that in each round of data transmission, the information size obtained after processing by the CH is *e* bit. The energy consumed in the process is calculated as follows:

(5) $$E_{CH}=(\frac{n}{k}-1)\cdot e\cdot E_{elec}+\frac{n}{k}\cdot e\cdot E_{DA}+e\cdot E_{elec}+\left\{ \begin{array}{l} e\varepsilon_{fs}d_{toBS}^{2},\;d_{toBS}<d_{0}\\ e\varepsilon_{mp}d_{toBS}^{4},\;d_{toBS}\geq d_{0} \end{array}\right.\;$$

In the above formula, the energy consumed consists of three parts: receiving energy consumption, processing energy consumption, and transmitting energy consumption. In Equation (5), *n* is the number of nodes surviving in the monitored area, *k* is the number of clusters to be divided, *E_DA_* is the energy consumed by the CH to process each bit of data (including received data and sensed data), and *d_toBS_* is the distance between the CH and the BS. 

### 2.3. Optimal Number of Clusters

In general, the inter-cluster communication traffic in WSN increases as the number of clusters increases, and the intra-cluster communication traffic increases as the number of clusters decreases. In addition, the network’s energy consumption increases as communication traffic increases. The determination of the optimal cluster number of the network is of great significance to the network’s communication. In this section, we will determine the optimal number of clusters *k* in combination with the network structure model and energy consumption model described respectively in Section 2.1 and Section 2.2.

The monitoring area in this paper is a circle with a diameter of *M*. In real life, the cluster areas of WSN must be irregular and inconsistent, and the nodes are randomly placed. If these three points are both considered, the proposed model must be complex and not universal. So like the optimal numbers of clusters in [11,12], that in this paper is also used as a relatively common reference standard in the actual monitoring area. To derive the optimal number of clusters more intuitively, we construct an inline square in the circular region with a side length of *L*. We assume that the clusters in the square are all circular in shape with a radius of *R* and the cluster distribution is uniformly distributed. Finally, after calculating the total circular cluster area and the circular area to establish the relationship, we can obtain the relationship between the total number of clusters *k* and the number of circular clusters *k*_1_.

**Figure 2:**
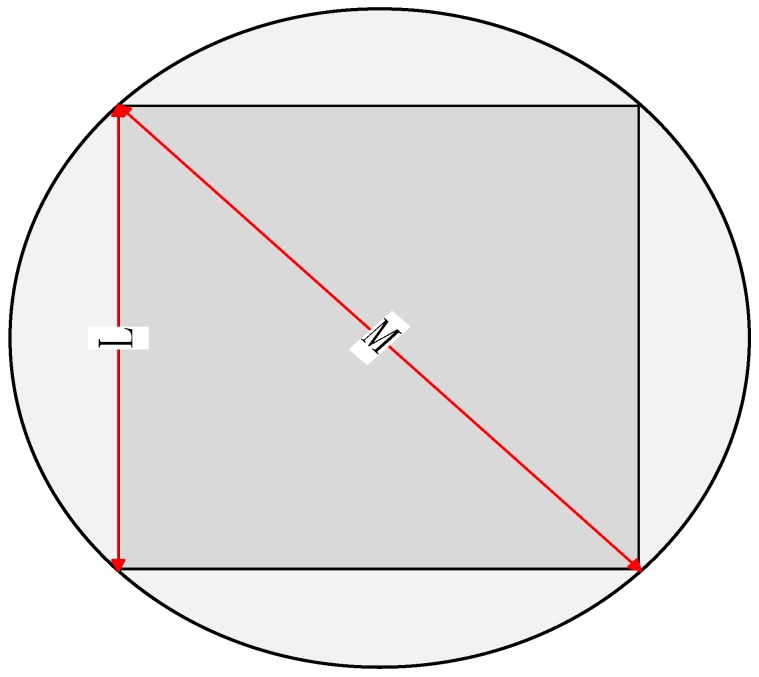
Monitoring area.

As shown in Figure 2, the monitoring area in this paper is a large circle with a diameter of *M* and the length of the embedded square is *L*. From this, we can derive the relationship between *L* and *M*:

(6) L=2M2 

After the sensor network is divided into many clusters, the CH receives the information transmitted by the cluster member nodes, and after processing, eventually transmits it to the BS for final data fusion. In the intra-cluster communication, as the distance between the cluster member node and the CH is not large, we adopt the free space model.

**Figure 3:**
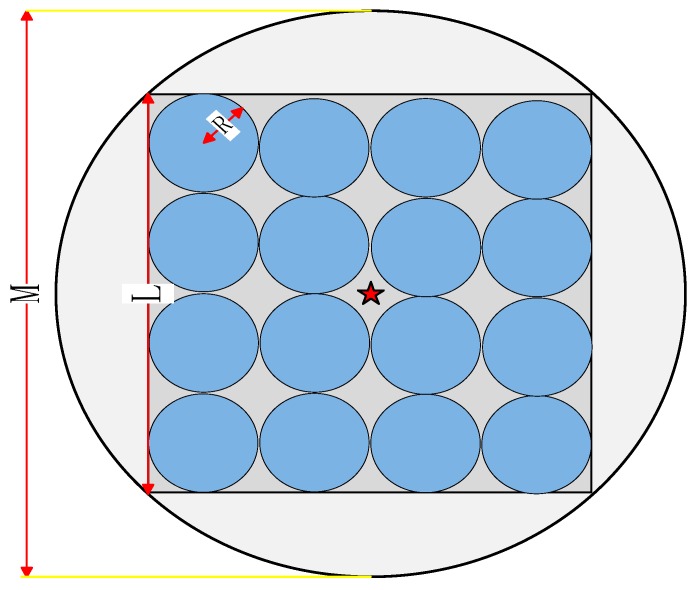
Network structure model diagram (16 clusters).

To understand the network structure model more intuitively, Figure 3 shows an example dividing the cluster into 16 clusters. In the figure, the positive center position of the monitoring area is the BS indicated by ☆. A blue circle indicates a cluster. Consequently, we can obtain the expression of the blue cluster number *k*_1_ as follows:

(7) $$k_{1}=\frac{M^{2}}{8R^{2}}\;$$

The area of the monitoring area *S_sum_* is calculated as follows:

(8) $$S_{sum}=\pi \cdot {\left(\frac{M}{2}\right)}^{2}=\frac{\pi M^{2}}{4}\;$$

The inscribed square area *S_square_* is calculated as follows:

(9) $$S_{square}={\left(\frac{\sqrt{2}}{2}M\right)}^{2}=\frac{M^{2}}{2}\;$$

One blue cluster area *S_cluster_* is calculated as follows:

(10) Scluster=πR2=π(22M2k1)2=πM28k1 

The total area of the blue clusters *S_cluster_sum_* is calculated as follows:

(11) $$S_{cluster\_sum}=k_{1}S_{cluster}=\frac{\pi M^{2}}{8}\;$$

According to Equations (8) and (11):

(12) $$S_{sum}=2S_{cluster\_sum}\;$$

If the cluster is completely divided into clusters in the monitoring area, the total number of clusters *k* is twice that of *k*_1_, namely:

(13) $$k=2k_{1}=\frac{M^{2}}{4R^{2}}\;$$

The cluster member nodes obey the uniform distribution, and then the distribution function can be expressed as follows:

(14) $$g(k)=\frac{8k_{1}}{\pi M^{2}}=\frac{4k}{\pi M^{2}}\;$$

Calculate the expected squared distance of the cluster member nodes to the CH.

(15) $$\begin{array}{l} E[d_{toCH}^{2}]=\int_{0}^{2\pi }d\theta \int_{0}^{R}g(k)\rho^{3}d\rho \\ \;=\int_{0}^{2\pi }d\theta \int_{0}^{R}\frac{4k}{\pi M^{2}}\rho^{3}d\rho \\ \;=\frac{2kR^{4}}{M^{2}} \end{array}$$

The distances between some CHs and BS in the model may be larger than *d*_0_, so it is necessary to simultaneously refer to the free space model and the multipath fading channel model when considering the energy consumption between clusters. Then the value range of the diameter *M* is greater than 2*d*_0_.

According to Equation (5):

(16) $$f(d_{toBS})=\left\{ \begin{array}{l} e\varepsilon_{fs}d_{toBS}^{2},\;d_{toBS}<d_{0}\\ e\varepsilon_{mp}d_{toBS}^{4},\;d_{toBS}\geq d_{0} \end{array}\right.\;$$

Then the expectation of *f*(*d_toBS_*) is calculated:

(17) $$\begin{array}{l} E[f(d_{toBS})]=E[f(\rho ,\theta )]\\ =\frac{4}{\pi M^{2}}\int_{0}^{2\pi }\int_{0}^{\frac{M}{2}}f(\rho ,\theta )\rho \;d\rho d\theta \\ =\frac{16e}{\pi M^{2}}\cdot \frac{\pi }{2}\left[\int_{0}^{d_{0}}\varepsilon_{fs}\rho^{3}\;d\rho +\int_{d_{0}}^{\frac{M}{2}}\varepsilon_{mp}\rho^{5}\;d\rho \right]\\ =\frac{2e\varepsilon_{fs}d_{0}^{4}}{M^{2}}+\frac{e\varepsilon_{mp}M^{4}}{48}-\frac{4e\varepsilon_{mp}d_{0}^{6}}{3M^{2}} \end{array}$$

(18) $$\mathrm{Let}\;A=\frac{2\varepsilon_{fs}d_{0}^{4}}{M^{2}}+\frac{\varepsilon_{mp}M^{4}}{48}-\frac{4\varepsilon_{mp}d_{0}^{6}}{3M^{2}}\;$$

(19) $$\mathrm{So}\;E[f(d_{toBS})]=A\cdot e\;$$

Thus, the average energy consumed by a cluster in one round is

(20) $$E_{cluster}=E_{CH}+(\frac{n}{k}-1)E_{non-CH}\approx E_{CH}+\frac{n}{k}E_{non-CH}\;$$

The energy consumed by all of the clusters in the region in one round is

(21) $$\begin{array}{l} E_{SUM}=kE_{cluster}=kE_{CH}+nE_{non-CH}\;=\;ne(E_{elec}+E_{DA})+kAe+neE_{elec}+ne\varepsilon_{fs}\cdot \frac{2kR^{4}}{M^{2}}\\ \;=ne(2E_{elec}+E_{DA})+kAe+\frac{M^{2}}{8k}\cdot ne\varepsilon_{fs} \end{array}$$

Deriving for *E_SUM_*, let $\frac{dE_{SUM}}{dk}=0$, so we can obtain the optimal number of clusters

(22) $$k=\sqrt{\frac{M^{2}n\varepsilon_{fs}}{8A}}\;$$

## 3. The Clustering Protocol

The main steps of the clustering protocol in this paper are as follows:

(1) Calculate the number *k* of clusters required according to the calculation formula of the network optimal cluster number introduced in Section 2.3.
(2) Through the AGNES (AGglomerative NESting) algorithm with balanced energy consumption optimization, we can build the required *k* clusters.
(3) Implement the selection mechanism of the CH in each cluster, then we can implement the D-CHs division of the energy balance strategy and node dormancy mechanism for the large cluster area before and after the death of the first node, respectively.
(4) Data transmission and energy update.

To minimize the total energy consumption and balance the energy consumption of the nodes in the network, we perform the node death decision after each round of data transmission in the network (once the node dies, return to Step 1; otherwise, return to Step 3). In addition, Steps 1, 2, and 3 are collectively called the preparation phase of the protocol. Step 4 is called the stabilization phase of the protocol. 

**Figure 4:**
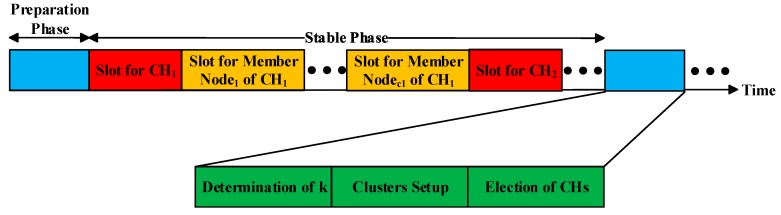
The time slot allocation of the clustering protocol.

**Figure 5:**
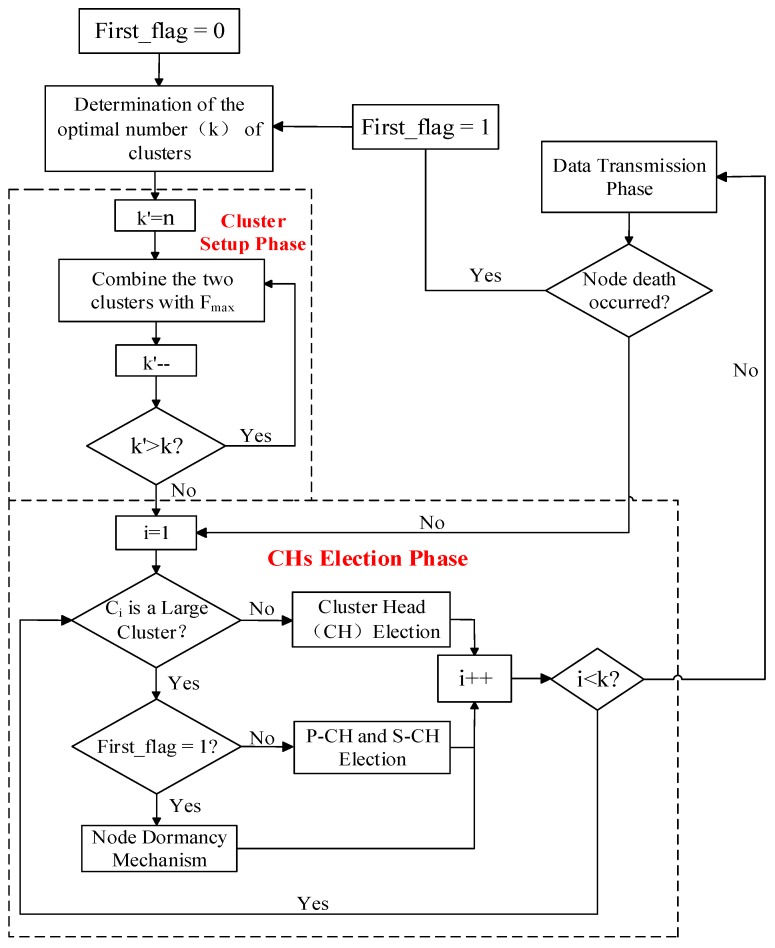
Flowchart of the clustering protocol, including the determination of *k*, cluster setup, cluster heads (CHs) election, and data transmission.

Before entering the stabilization phase, each member of each cluster needs to send a set of control message named as *Node_Msg* to its CH in the form of (*Node_NO*, *Node_Status*). The status is only divided into work and dormancy. According to the received *Node_Msg*, the CH of each cluster allocates a time slot for the member nodes of the cluster that need to work. Then every CH sends a set of control message named as *Schedule_Msg* to its member nodes that need to work in the form of (*Node NO.1*, *Time Slot1*; *Node NO.2*, *Time Slot2*; ……). Once entering the stabilization phase, the nodes which have received time slots send their sensed information to their associated CHs, and others are in dormancy. As for the CHs, they are responsible for receiving and processing the information sent by the member nodes and eventually transmitting it to the BS. The time slot allocation of the clustering protocol in this paper is provided in Figure 4. A flowchart of the clustering protocol in this paper is presented in Figure 5. 

### 3.1. AGNES Algorithm

The AGNES (AGglomerative NESting) [30] algorithm is a hierarchical clustering algorithm. First, several objects are input, each one constitutes an initial cluster by itself. Then the two clusters with the shortest distance are continuously merged into one cluster until the number of clusters obtained reaches the number of clusters *k* satisfying the termination condition. Finally, the resulting *k* clusters are the target clusters of our algorithm. 

In this algorithm, each cluster equals a sample set, and the merger between clusters equals the merger between sets. The merging standard is the distance between the two clusters, which usually assumes three forms: (1) the longest distance, (2) the shortest distance, and (3) the average distance. For example, given two clusters (*C_i_* and *C_j_*), the distance between the two clusters can be obtained from the following three equations:

The longest distance:

(23) $$D_{\max}(C_{i},C_{j})=\underset{p\in C_{i},q\in C_{j}}{\max}\left|p-q\right|\;$$

The shortest distance:

(24) $$D_{\min}(C_{i},C_{j})=\underset{p\in C_{i},q\in C_{j}}{\min}\left|p-q\right|\;$$

The average distance:

(25) $$D_{avg}(C_{i},C_{j})=\frac{1}{\left|C_{i}\right|\left|C_{j}\right|}\sum_{p\in C_{i}}\sum_{q\in C_{j}}\left|p-q\right|\;$$

### 3.2. The AGNES Algorithm with Balanced Energy Consumption Optimization

To further improve the various indicators of WSN, we make balanced energy consumption optimization for the AGNES algorithm:

(1) To reduce the difference in the distance set between the nodes in the two clusters: On the basis of the original indicator that can be combined in two clusters (note: the average distance *D_avg_* is used in this paper), the distance set variance *δ*^2^ is added, so the two merged clusters cannot only have a shorter average distance, but also the distance difference between the nodes in the two clusters tends to be smaller. Thus, the energy consumption of the cluster nodes is more uniform, which can help effectively avoid the phenomenon that some cluster member nodes die prematurely due to the jaggedness of the transmission distances during the communication process.
(2) The D-CHs division of the energy balance strategy in large clusters is implemented before the death of the first node: The AGNES algorithm can obtain the *k* cluster needed, but it does not limit the size of the cluster, so the resulting clusters may have different sizes. As a result, CHs in large clusters tend to receive and process large amounts of cluster information, then the energy is prematurely exhausted, which will have a very negative impact on network lifetime extension, energy consumption reduction, and throughput increase. Based on this, we implement the D-CHs division of energy balance strategy in the large cluster area before the death of the first node. The strategy mainly includes the following: the secondary cluster head (S-CH) is responsible for receiving the information sent by the cluster member nodes and the positive cluster head (P-CH) is responsible for merging the former information with the self-sensing information and finally transmitting it to the BS.
(3) The node dormancy mechanism in large clusters is implemented after the death of the first node as follows: The data obtained by WSN needs to meet two requirements (large amount of data and high data integrity). Before the first node dies, the network is in a stable period, and the energy of the node group is enough, and many rounds of iterations can be performed. At this time, the data in the network can well satisfy the above two requirements. The node dormancy mechanism will cause data loss in some areas while causing energy consumption reduction. Therefore, the node dormancy mechanism is not implemented at this time. But after the death of the first node, it means that the energy of the node is greatly reduced, and the mortality rate is greatly improved. Even if the node dormancy mechanism is not performed, the network coverage of the monitoring area becomes smaller as the nodes die continuously. It will inevitably lead to a reduction in data integrity. At this time, it is not practical to maintain data integrity as well as the stable period. Therefore, our focus is on the improvement of data volume. By extending the network life cycle, WSN will have a longer monitoring time for the region, and can obtain a larger amount of information. The node dormancy mechanism can make the nodes which have relatively low energy in the cluster and relatively long distance from the CHs be in dormancy, avoiding its premature death, and reducing the energy load of the cluster head, thereby prolonging the network life cycle, which just satisfies the actual needs of the period.

It’s worth emphasizing that the protocol in this paper must re-select the CHs at the end of each round, which can help balance the energy consumption of the nodes and maintain the network coverage in the area. 

### 3.3. Cluster Setup

By adding the two cluster average distance *D_avg_*, and the variance *δ*^2^ of the distance set of two clusters in the cluster setup process, we can construct a cluster setup factor. The two clusters corresponding to the largest cluster-merging factor can be merged until the number of clusters reaches the pre-set number of clusters *k*. Compared to the original AGNES algorithm, the algorithm has smaller distance difference between the nodes in the two clusters, and therefore the energy consumption of the nodes is more uniform during data transmission. The detail procedure of this phase is given by the pseudo-code in Algorithm 1.

**Algorithm 1.** Cluster Setup Algorithm**Inputs:** (1) *n* objects     (2) *k—*number of clusters**Result:***k* clusters of different sizes1: Each object constitutes an initial cluster;2: Current cluster number *k’* = *n*;3: while (*k*’ > *k*)4:   for *i* = 1 → *k’*5:     for *j* = 1 → *k’*6:       if *i* ~= *j*7:         calculate the distance of the two nodes in the two clusters *C_i_* and *C_j_*;8:         build a distance set *D* and obtain the distance mean *D_avg_* and the variance *δ*^2^;9:         calculate the clustering factor of clusters *C_i_* and *C_j_*$F_{i,j}(D_{avg},\delta^{2})=\frac{1}{D_{avg}+0.5\delta^{2}}$
10:     end if11:   end for12: end for13: combine the two clusters corresponding to *F_max_*14: *k’*--;15: end while

### 3.4. CHs Election

This section is mainly divided into two parts: (1) CHs election in general clusters; and (2) CHs election in large clusters. 

#### 3.4.1. CHs Election in General Clusters

In general, if a node in a cluster wants to be the CH of the cluster, the following three conditions must be met:

(a) Compared to most other nodes, its location is closer to the center of the cluster.
(b) Compared to most other nodes, its distance from BS is relatively small.
(c) Compared to most other nodes, it owns more residual energy.

On this basis, we calculate the center position *Cen* (*X_C_*, *Y_C_*) of a cluster by the following formula:

(26) $$X_{C}=\frac{\sum_{p\in C}X_{p}}{\left|C\right|}\;$$

(27) $$Y_{C}=\frac{\sum_{p\in C}Y_{p}}{\left|C\right|}\;$$

where *|C|* represents the number of nodes in the cluster *C*. 

Then we construct an objective function for picking an appropriate node as the CH in cluster *C*.

(28) $$\;G_{CH}\left(S\left(i\right)\cdot E,d_{toC\mathrm{en}}^{i},d_{toBS}^{i}\right)=\frac{S\left(i\right)\cdot E}{\alpha d_{toCen}^{i}+\beta d_{toBS}^{i}}\;$$

In Equation (28), *S*(*i*).*E* represents the residual energy of node *i*; *d^i^_toCen_* represents the distance from node *i* to *Cen*; *d^i^_toBS_* represents the distance from node *i* to the BS; and *α* and *β* are respectively the routing factors of *d^i^_toCen_* and *d^i^_toBS_*, where *α +* *β* = 1. The larger the value of *G_CH_* of node *i*, the more likely it is to be selected as the CH. Algorithm 2 shows us the CH election in a general cluster.

**Algorithm 2.** CH Election Algorithm (General Cluster)**Inputs:** (1) nodes of cluster *C*     (2) size of cluster C: *NO*     (3) location of BS: (*BS·x*, *BS·y*)**Result:** CH of Cluster *C*1: calculate cluster center position *Cen* (*X_C_*, *Y_C_*);2: for *i* = 1 → *NO*3:   calculate the objective function of node *i*, *G^i^_CH_*4: end for5: select the node with *GCH_max_* as the CH of cluster *C*;

#### 3.4.2. CHs Election in Large Clusters

For large clusters, before the death of the first node, we implement the D-CHs division of energy balance strategy, which involves the selection of the positive cluster head (P-CH) and the secondary cluster head (S-CH). The detail procedure is given by the pseudo-code in Algorithm 3. In this paper, a cluster with CH energy consumption greater than 1.5 times the average CH energy consumption is defined as a large cluster. According to the energy consumption formulas introduced in Section 2.2 and Section 2.3, we can calculate the average energy consumption of the CH, $\bar{E_{CH}}$.

(29) $$\begin{array}{l} \bar{E_{CH}}=\left(\frac{n}{k}-1\right)\cdot e\cdot E_{elec}+\frac{n}{k}\cdot e\cdot E_{DA}+e\cdot E_{elec}+E[f(d_{toBS})]\\ \;=\frac{n}{k}\cdot e(E_{elec}+E_{DA})+A\cdot e \end{array}$$

We use *x* to represent the total number of nodes in the cluster, so we can use the following formula to get the CH energy consumption *E* of the cluster.

(30) $$\begin{array}{l} E=(x-1)eE_{elec}+xeE_{DA}+eE_{elec}+E[f(d_{toBS})]\\ \;=xe(E_{elec}+E_{DA})+A\cdot e\; \end{array}$$

Next, we determine what the value of *E* is when the corresponding cluster can be defined as a large cluster. Here, we assume that *E* is *γ* times as $\bar{E_{CH}}$. We compare the difference between the energy consumption of the CH in the cluster and the average energy consumption of the CH in the case of General CHs and D-CHs, respectively, to obtain the critical *E* value of a large cluster.

**Figure 6:**
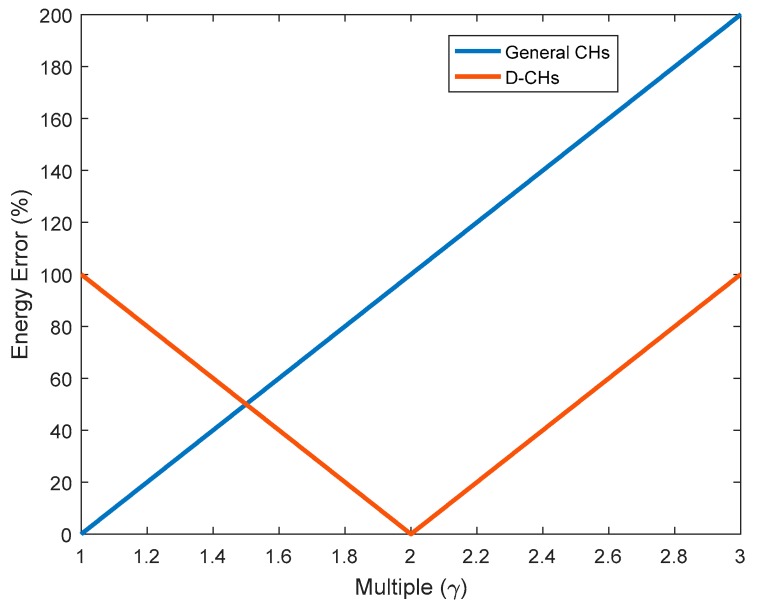
A comparison of energy error between General CHs and dual-cluster heads (D-CHs).

According to Figure 6, when *γ* is less than 1.5, the energy error of General CHs is less than D-CHs; Once *γ* is more than 1.5, the energy error of D-CHs is less than General CHs, which means the effect of D-CHs division of energy balance strategy is better than General CHs strategy. So, we determine the value of *γ* is 1.5.

To compute $E>1.5\times \bar{E_{CH}}$, it can be concluded that the number of nodes in the large cluster is required to satisfy the condition:

(31) $$x>1.5\frac{n}{k}+0.5\frac{A}{E_{elec}+E_{DA}}\;$$

**Figure 7:**
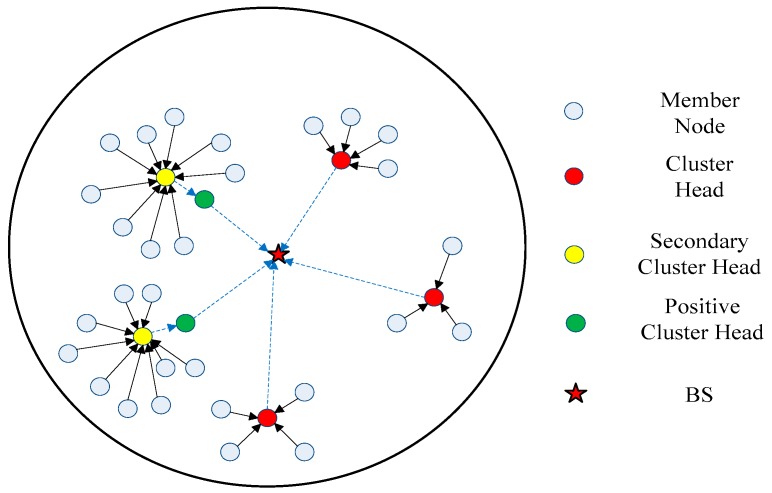
WSN clustering topology (D-CHs division of energy balance strategy in large clusters).

**Algorithm 3.** P-CH and S-CH Selection Algorithm (Large Cluster of Stable Period)**Inputs:** (1) nodes of cluster *C*     (2) size of cluster C: *NO*     (3) location of BS: (*BS·x*, *BS·y*)**Result:** P-CH and S-CH of cluster *C*1: calculate cluster center position *Cen* (*X_C_*,*Y_C_*);2: for *i* = 1 → *NO*3:   calculate the objective function of node *I*, *G^i^_CH_*;4: end for5: select two nodes with *GCH_max_* and *GCH_second-max_* as the P-CH and S-CH of cluster *C*, respectively;

As shown in Figure 7, the WSN consists of 5 clusters (2 large clusters and 3 general clusters). Compared to the general cluster, the member nodes (white dots) in the large cluster collect environmental information and send it to the S-CH (yellow dots); the latter receives such information and the data fusion is performed in the P-CH (green dots). The final information is sent to the BS (red pentagram) for decision making.

### 3.5. Node Dormancy Mechanism

In general, the node dormancy mechanism is mainly divided into two categories: (1) randomly select a certain proportion of nodes to be dormant based on their different locations and (2) select nodes of different proportions to be dormant based on their distance to the CHs. 

After the network enters the unstable period, the cluster member nodes in the large cluster area have low energy and long transmission distance, so it is extremely easy for them to exhaust their energy. The CH needs to receive and process a large amount of information, and once it dies, all of the information in the cluster cannot be transmitted to the BS, so that valuable information may be lost. 

Based on the residual energy of the cluster member nodes and their distances to the CHs, the node dormancy mechanism proposed in this paper causes the member nodes with low energy and long distances to the CH to become dormant, thus reducing the load on the CH and improving the network throughput. Its steps are as follows: 

First, set up dormancy factors *S_dor_* for all of cluster member nodes, which is calculated as follows:

(32) $$\;S_{dor}\left(S\left(i\right)\cdot E,d_{toBS}^{i}\right)=\frac{S\left(i\right)\cdot E}{d_{toBS}^{i}}\;$$

The smaller the *S_dor_* of the node *i*, the higher the mortality rate of the node *i* and the higher the dormancy probability. 

Next, the node dormancy ratio *P* is determined.

(33)  P=(C(j)⋅NO−nk)/C(j)⋅NO 

In Equation (33), *C*(*j*)*·NO* represents the total number of nodes in cluster *j*, *n* is the number of currently surviving nodes, and *k* is the number of clusters established. 

Finally, the dormancy factors of all of the cluster member nodes in the large cluster are sorted from small to large, then the nodes corresponding to the pre-*P* dormancy factors are dormant. The detail procedure is given by the pseudo-code in Algorithm 4.

To understand the node dormancy mechanism more intuitively, look at Figure 7 (A simple example). 

**Figure 8:**
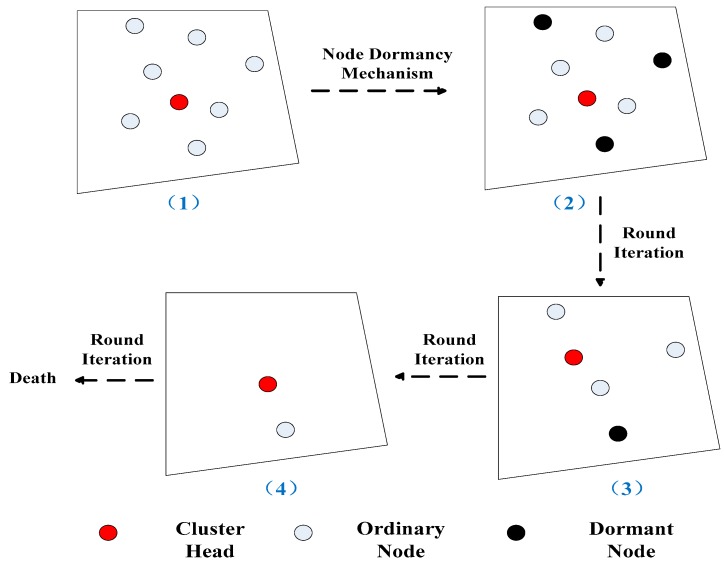
Node dormancy diagram of a cluster.

As shown in Figure 8, first, after the CH is determined in (1), the node dormancy mechanism is implemented. Then the three dormant nodes are determined in (2). After several rounds of data iterations, we determined a new CH and a dormant node in (3). After several further rounds of data iterations, only two nodes survive in (4). After re-determining the CH and the last multiple rounds of data iterations, all of the nodes of the cluster die.

**Algorithm 4.** Node Dormancy Algorithm (Large Cluster of Unstable Period)**Inputs:** (1) nodes of cluster *C*     (2) size of cluster C: *NO*     (3) number of surviving nodes: *n*     (4) optimal number of clusters: *k***Result:** dormant nodes of cluster *C*1: for *i* = 1 → *NO*2:   calculate the dormancy factor of node *i*, *S^i^_dor_*3: end for4: calculate node dormancy ratio, *P*5: sort the dormancy factor set from small to large6: put the nodes corresponding to the previous *P* dormancy factors into dormancy

## 4. Simulation Results

In this section, we evaluate the proposed protocol by simulating using MATLAB 2016b (MathWorks, Natick, MA, USA) on a desktop PC (Lenovo, made in Beijing, China) with Intel(R) Core (TM) i3-4170 CPU @ 3.70GHz, 4GB RAM. When building the network model, this study assumed that all of the wireless sensor nodes are distributed in a circular area with a diameter of *M*, and the BS is located at the center of the area (0, 0). Specific parameters in the simulation are shown in Table 1 (Note that J in this paper stands for Joule, which is a unit of energy). We mainly compared our proposed protocol to the original classic protocols from the two kinds of networks including the homogeneous and heterogeneous networks, and the four aspects including the death round of the first node, the lifetime of the network, the trend of the network energy consumption, and the trend of the network throughput with the rounds of iterations. 

### 4.1. Determination of the Optimal Routing Factor

Based on the CHs priority function mentioned in Section 3.4, we can select the nodes with better positions and more adequate residual energy as the CHs. To get the optimal routing factor α, we conduct related simulations, including α is taken from 9 numbers between 0.1 and 0.9 and the network lifetime is simulated and compared in homogeneous networks (initial energy = 0.6 J) and heterogeneous networks (the initial energy is evenly distributed at 0.4–0.8 J).

#### 4.1.1. The Network Lifetime Comparison in Homogeneous Networks

**Figure 9:**
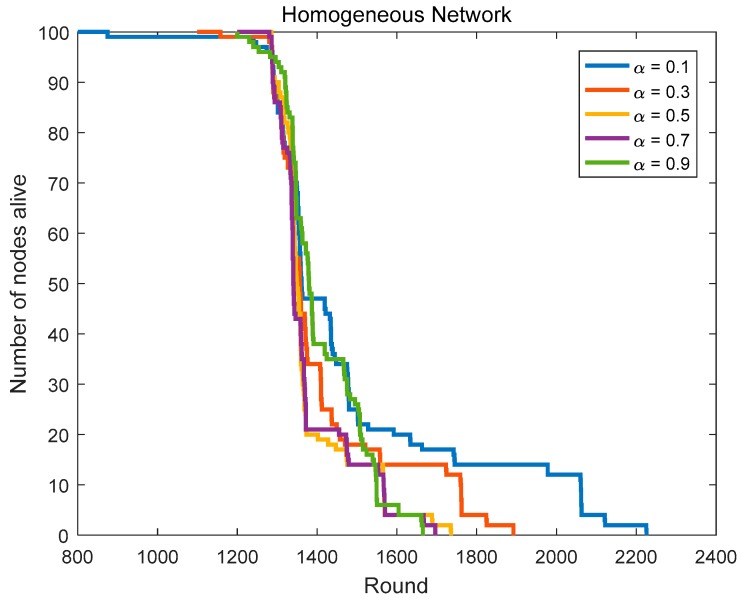
The network lifetime comparison in homogeneous networks (routing factor = 0.1, 0.3, 0.5, 0.7 and 0.9).

**Figure 10:**
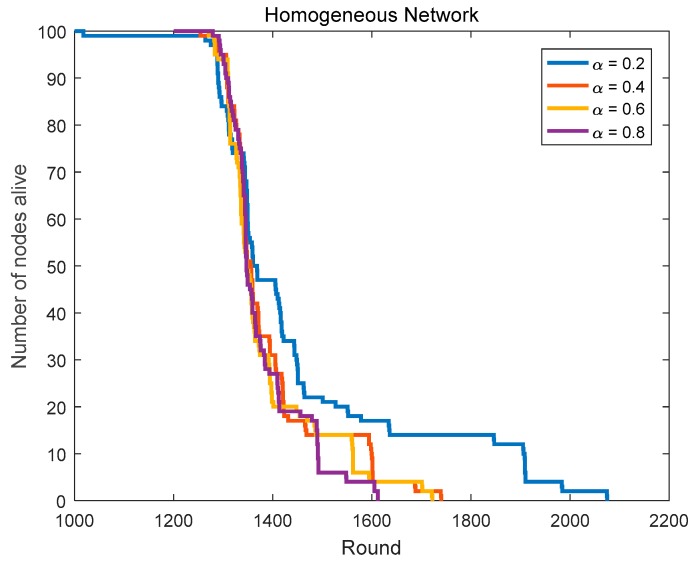
The network lifetime comparison in homogeneous networks (routing factor = 0.2, 0.4, 0.6 and 0.8).

In Section 4.1.1, we compare the network lifetime in homogeneous networks with α taking from 9 numbers between 0.1 and 0.9. Figure 9 and Figure 10, and Table 2 show us the related results.

#### 4.1.2. The Network Lifetime Comparison in Heterogeneous Networks

**Figure 11:**
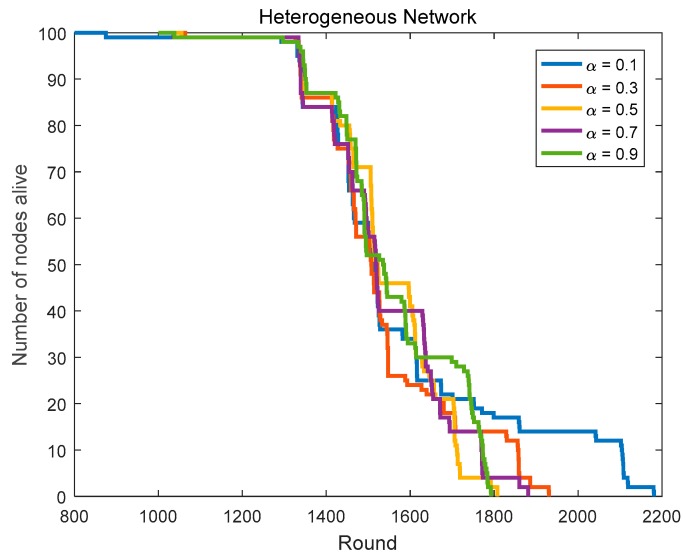
The network lifetime comparison in heterogeneous networks (routing factor = 0.1, 0.3, 0.5, 0.7 and 0.9).

**Figure 12:**
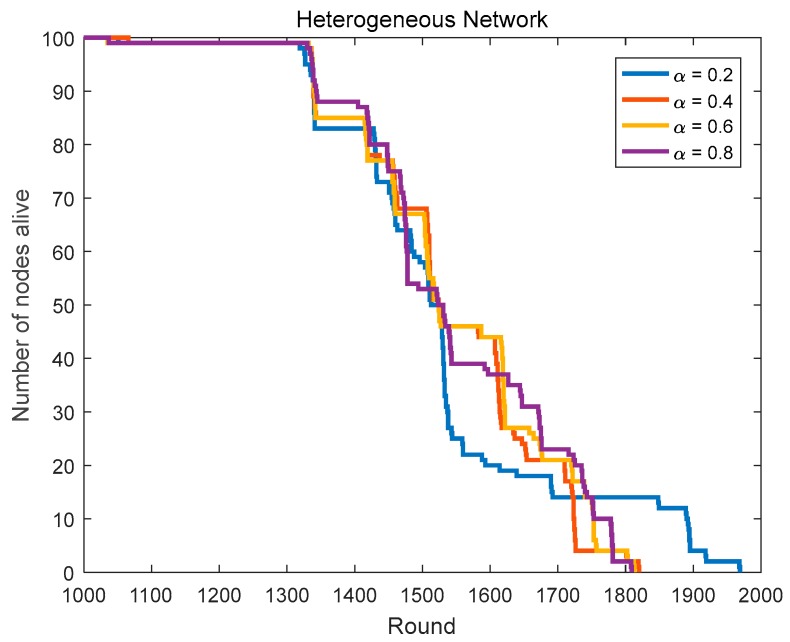
The network lifetime comparison in heterogeneous networks (routing factor = 0.2, 0.4, 0.6 and 0.8).

In Section 4.1.1, we compare the network lifetime in heterogeneous networks with α taking from 9 numbers between 0.1 and 0.9. Figure 11 and Figure 12, and Table 3 show us the related results.

#### 4.1.3. The Optimal Routing Factor

Although the network has the longest lifetime in the case of α = 0.1 compared with other cases, its first node death time is too early, which means that the energy consumption distribution of the nodes is rather uneven in this case. In homogeneous networks, the first node’s death round in the case of α = 0.2 is 1018, it is too small. Although the first node’s death rounds in the case of α = 0.4–0.9 are very close, the last node’s death round in the case of α = 0.4 is the largest among them. The first node’s death round in the case of α = 0.3 is less than that in the case of α = 0.4, but the last node’s death round in the case of α = 0.3 is more than that in the case of α = 0.4. Thus, the optimal routing factor α in homogeneous networks is 0.3 or 0.4.

In heterogeneous networks, the first node’s death rounds in the case of α = 0.2–0.9 are very close, but the last node’s death rounds in the case of α = 0.2 and 0.3 are both the largest among them. Thus, the optimal routing factor α in heterogeneous networks is 0.2 or 0.3.

In summary, we can determine the optimal routing factor (α = 0.3) in the protocol.

### 4.2. Comparison of the Death Round of the First Node

In WSNs, network performance tends to decline with the nodes’ death, and the network is in a stable period before the first node dies. The death of the first node indicates that the network enters an unstable period and its performance starts to decline. The clustering protocol in this paper balances the energy consumption of the network by cyclically selecting the CHs and considers the remaining energy and location of the node in the process of the CHs selection. 

**Figure 13:**
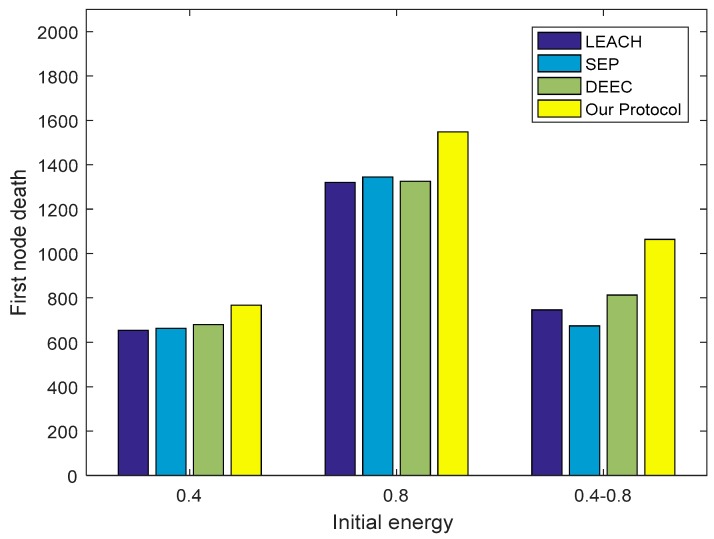
The round of the first node’s death. LEACH: Low-Energy Adaptive Cluster Hierarchical; SEP: Stable Election Protocol; DEEC: Distributed Energy-Efficient Clustering.

Figure 13 shows that in the homogeneous networks (0.4 J and 0.8 J), the round of the first node’s death in the three protocols LEACH, SEP, and DEEC is not substantially different. In comparison, our protocol has an advantage in delaying the round of the first node’s death. In the heterogeneous networks (0.4–0.8 J), our protocol can still maintain good performance in this respect. At this time, the DEEC performance is the best among the other three protocols, the LEACH performance is second, and the SEP performance is poor. It is not difficult to understand that in heterogeneous networks, the energy gap between nodes is large. But regardless of the energy of the nodes in the network, the same probability that LEACH gives these nodes is elected as the CH. SEP only considers the initial energy of the node that will cause the high-energy node to have less energy but maintain a high probability of being selected as the CH after multiple rounds of iterations, so that it increases the death rate of the node and causes the first node to die the earliest. DEEC comprehensively analyzes the initial energy and residual energy of the node, which can ensure the probability that the node with high initial energy is elected as the CH can be lowered after multiple rounds of data iterations, so that other nodes with high remaining energy have higher probability to be elected as the CH. The commonality of the three protocols is that they do not consider the location of the node, resulting in some nodes with much energy and relatively remote locations being elected as the CHs, thus causing unnecessary energy waste. Therefore, the protocol in this paper considers the energy and position of the node, so that the CH in one round tends to have more energy and better position, thus effectively extending the death round of the first node.

### 4.3. Comparison of the Network Lifetime

**Figure 14:**
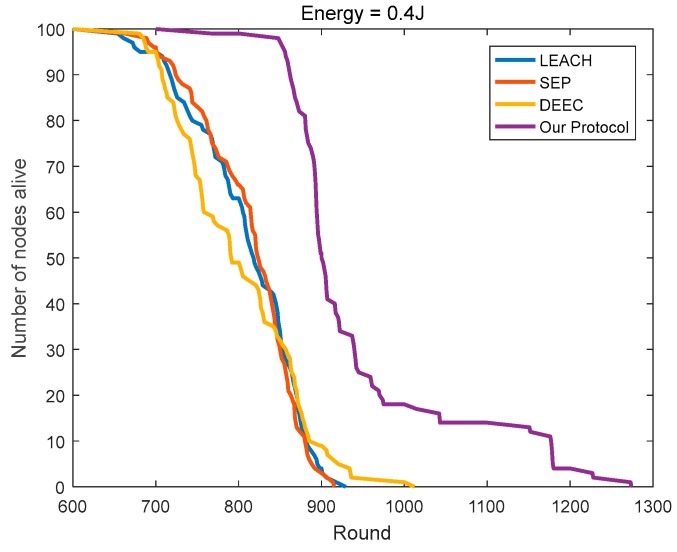
The number of surviving nodes varies with the round (initial energy = 0.4 J).

**Figure 15:**
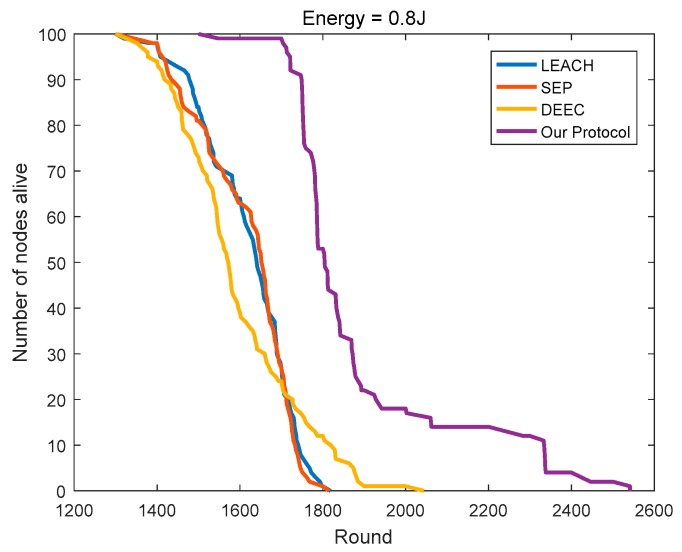
The number of surviving nodes varies with the round (initial energy = 0.8 J).

SEP considers the impact of the initial energy on the basis of LEACH. Yet in the homogeneous networks, the initial energy of all of the nodes is the same, then SEP equals LEACH. As shown in Figure 14 and Figure 15, the numbers of surviving nodes with the rounds of SEP and LEACH are very close, which is a good testimony to its efficacy. With the continuous rounds of iterations, the advantage of DEEC gradually emerged. Compared to LEACH, DEEC extends the network lifetime by 8.93% and 12.37% in the two homogeneous networks, respectively. Compared to DEEC, the protocol in this paper further extends the network lifetime by 25.89% and 24.36%, respectively.

**Figure 16:**
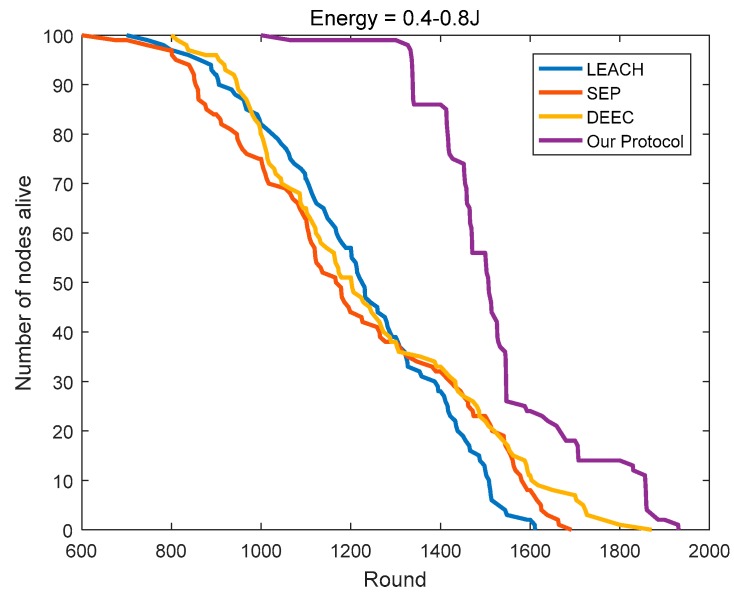
The number of surviving nodes varies with the round (the initial energy is evenly distributed at 0.4–0.8 J).

As shown in Figure 16, in heterogeneous networks, compared to LEACH, the number of surviving nodes in SEP is less than that in LEACH in the early period. SEP causes many nodes with high initial energy to die in the early period, so it has more nodes with less initial energy in the network. During the later period, the energy distribution of the nodes in SEP is more balanced so that it can maintain a longer network lifetime. Considering the initial energy and residual energy of the node, DEEC has advantages in heterogeneous networks compared to SEP and LEACH. Compared to LEACH, SEP, and DEEC, the protocol in this paper leads to the survival of 86 nodes in the 1400th round, thus ensuring that the protocol can carry more rounds of network communication, while LEACH, SEP, and DEEC retain only 28, 32, and 33 nodes, respectively. 

### 4.4. Comparison of the Network Energy Consumption

**Figure 17:**
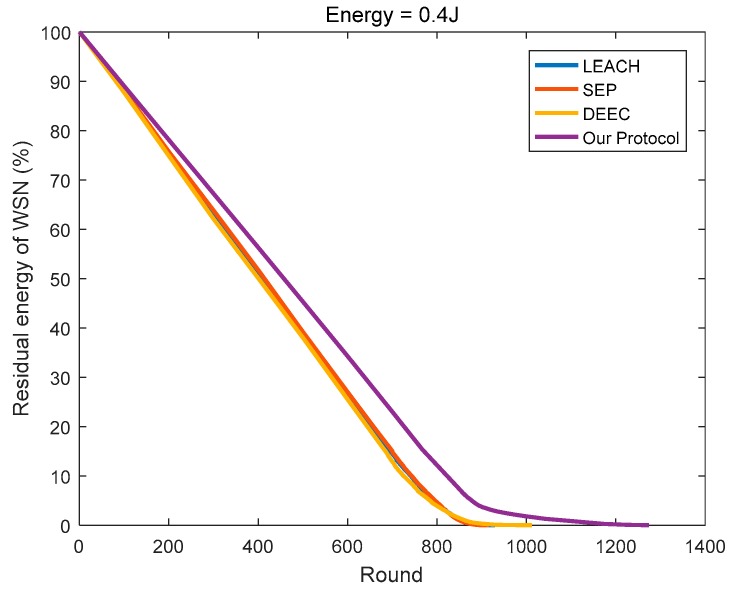
The residual energy varies with the round (initial energy = 0.4 J).

**Figure 18:**
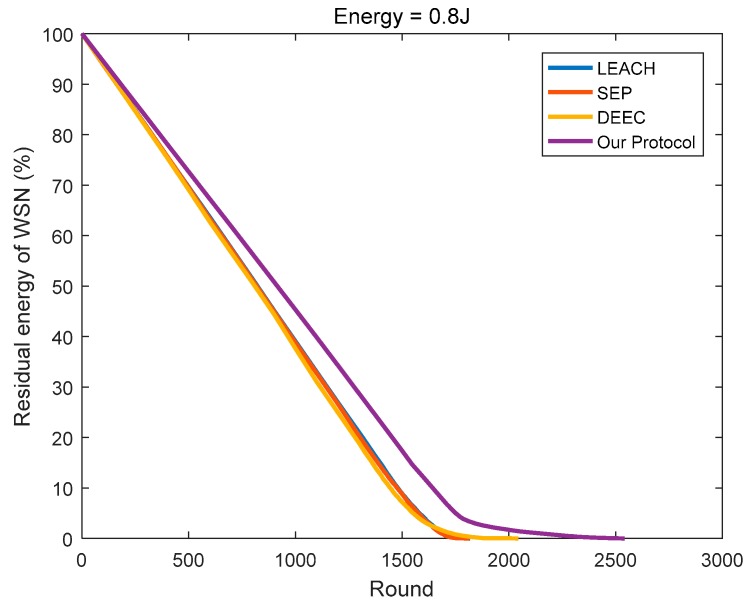
The residual energy varies with the round (initial energy = 0.8 J).

**Figure 19:**
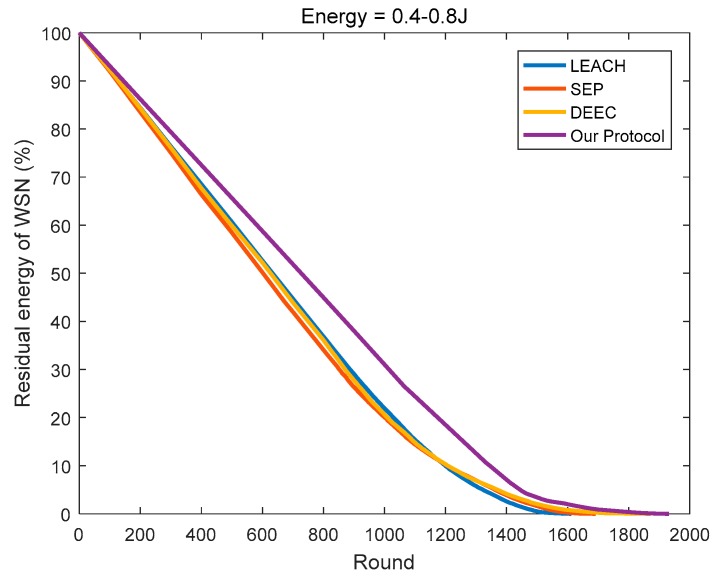
The residual energy varies with the round (the initial energy is evenly distributed at 0.4–0.8 J).

In this paper, the energy consumption model proposed in [12] is referenced in determining the optimal cluster number of the network, and a new optimal cluster number method is proposed according to the specific network model. Then, for each round of data transmission, we determine the most suitable CH based on the remaining energy and positions of the nodes in the cluster. As shown in Figure 17, Figure 18, Figure 19, compared to the other three protocols, the number of clusters calculated by the protocol in this paper is superior, and the CHs selection mechanism is more reasonable, so less energy is consumed in the network.

### 4.5. Comparison of the Network Throughput

Network throughput is an important indicator that fundamentally reflects the performance of a protocol. It refers to the number of packets in the network that are ultimately sent to the BS. The cluster member nodes transmit the information sensed by itself to the CH in the form of packets, and the CH fuses this information with that sensed by itself, and finally sends the information to the BS in the form of packets. During this period, if the energy of the CH is insufficient to receive, fuse, or transmit the information, all of the information of the cluster in this round cannot be transmitted to the BS, resulting in a decrease in network throughput. 

**Figure 20:**
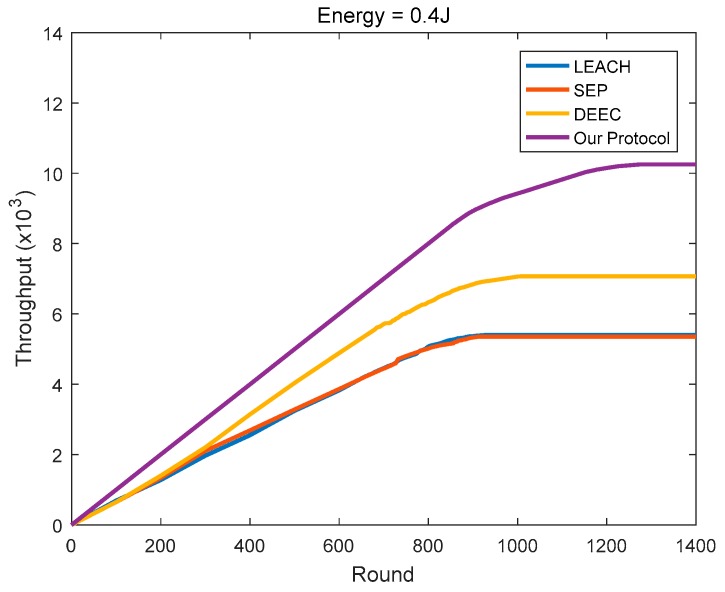
The network throughput varies with the round (initial energy = 0.4 J).

**Figure 21:**
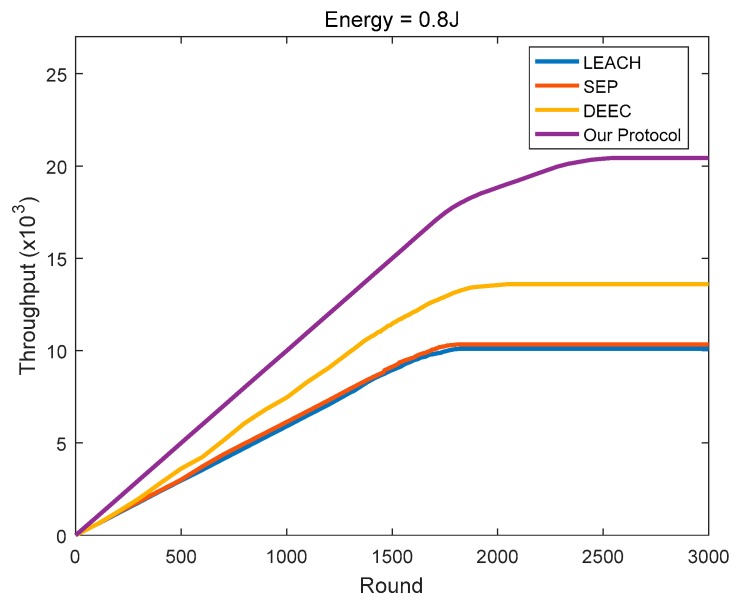
The network throughput varies with the round (initial energy = 0.8 J).

**Figure 22:**
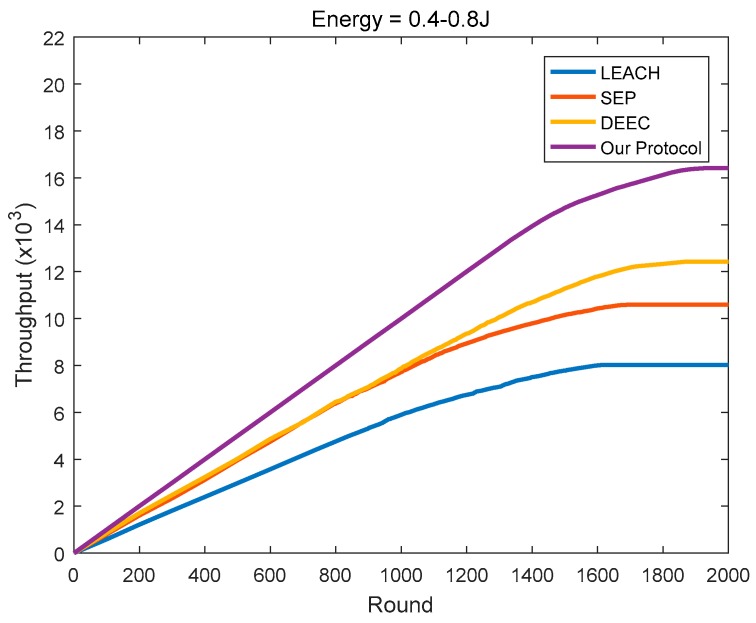
The network throughput varies with the round (the initial energy is evenly distributed at 0.4–0.8 J).

As shown in Figure 20, Figure 21 and Figure 22, the protocol in this paper achieves a good improvement in the network throughput: 

In the homogeneous networks with the initial energy of 0.4 J, the final throughputs of LEACH, SEP, and DEEC are 5404, 5357, and 7072, respectively. In comparison, the protocol in this paper achieves 89.64%, 91.3%, and 44.9% in throughput improvement, respectively. 

In the homogeneous networks with the initial energy of 0.8 J, the final throughputs of LEACH, SEP, and DEEC are 10,099, 10,336, and 13,590, respectively. In comparison, the protocol in this paper achieves 102.32%, 97.68%, and 50.35% in throughput improvement, respectively. 

In the heterogeneous networks where the initial energy is evenly distributed at 0.4–0.8 J, the final throughputs of LEACH, SEP, and DEEC are 8017, 10,591, and 12,426, respectively. In comparison, the protocol in this paper achieves 104.79%, 55.02%, and 32.13% in throughput improvement, respectively. 

As indicated in Table 4, Table 5, Table 6, Table 7 and Table 8, as the best among the four clustering protocols, our protocol can achieve a longer first node’s death round, longer network lifetime, lower energy consumption and a higher amount of communication data than the others, which is of great significance for various environmental monitoring applications.

## 5. Conclusions

To further improve the performance of WSNs and increase the application value of WSNs in various scenarios, this paper proposes a new WSN clustering routing protocol. First, a new network structure model is introduced, then according to the original energy consumption model, a new method for determining the optimal cluster number of the network is proposed to balance the energy consumption within the cluster and between the clusters. Next, aiming at the shortcomings of the original AGNES algorithm, this paper introduces the variance in the cluster setup phase to reduce the difference in the distance between the nodes in the two clusters and implements the D-CHs division of the energy balance strategy and the node dormancy mechanism before and after the death of the first node, respectively. Finally, the CH priority function is constructed based on the residual energy and position of the node and the CHs are selected repeatedly at the end of each round. The simulation results show:

In homogeneous networks, the performance of LEACH and SEP is similar to each other. At this time, the rounds of the first node’s death in the three protocols LEACH, SEP, and DEEC are not substantially different. The protocol in this paper has increased by approximately 13–17% in the round of the first node’s death. Compared to LEACH and SEP, the protocol in this paper has increased by approximately 40% and 90–103% in network lifetime and network throughput, respectively; Compared to DEEC, the protocol in this paper has increased by approximately 25% and 45–50% in network lifetime and network throughput, respectively.

In heterogeneous networks, compared to LEACH, the advantages of SEP and DEEC are gradually reflected. In the stable period, SEP causes many nodes with high initial energy to act as CHs frequently, so the first node’s death round in SEP is less than that in LEACH. Different from the former two, DEEC considers the residual energy of the nodes, so that the energy consumption of the nodes in the network is relatively more balanced. Compared with LEACH and SEP, its overall network performance is better. For the protocol in this paper, it takes into account the location and the remaining energy of the node, so that the total energy consumption in the network is smaller and the energy consumption is more balanced, and the network can survive more nodes after multiple iterations than the other three. Especially in terms of network throughput, it has increased by approximately 32% than DEEC.

So, the protocol can achieve a certain improvement in terms of the round of the first node’s death, network lifetime, network energy consumption, and network throughput.

However, there are some shortcomings in our protocol: 

First, the scenario considered by the protocol is too idealistic. In reality, even if the node energy is sufficient, transmission failure may occur due to the uncertainty of the natural environmental factors in the information transmission process. We can later consider adding a probability model to simulate the natural environment during the information transmission process. 

Second, the protocol is applicable only to 2D scenarios. Typical 3D scenarios, such as underground coal mines, underground pipe corridors, and indoor homes, in which, WSN must be arranged in three dimensions. Therefore, in the future we will consider proposing a clustering routing protocol suitable for 3D scenarios based on this protocol.

Last, the protocol proposed in this paper does not optimize the information transmission path. Therefore, compared with some general low-latency protocols, its delay may be larger, which is not suitable for some projects with higher real-time requirements. Therefore, in the future we will consider optimizing the information transmission path through a relatively practical optimization algorithm. 

## Figures and Tables

**Table 1:** Simulation parameters.

Parameter	Value
*E_elec_*	50 nJ/bit
*E_DA_*	5 nJ/bit/message
*ε_fs_*	10 pJ/bit/m^2^
*ε_mp_*	0.0013 pJ/bit/m^4^
The diameter of monitoring area, *M*	200 m
Initial number of nodes, *N*	100
Size of message, *e*	4000 bits
Initial energy	0.4~0.8 J

**Table 2:** A comparison of the first and last node’s death round in homogeneous networks with different routing factor α.

Routing Factor α	The First Node’s Death Round	The Last Node’s Death Round
0.1	875	2226
0.2	1018	2075
0.3	1158	1892
0.4	1254	1740
0.5	1285	1736
0.6	1271	1722
0.7	1280	1696
0.8	1279	1612
0.9	1200	1665

**Table 3:** A comparison of the first and last node’s death round in heterogeneous networks with different routing factor α.

Routing Factor α	The First Node’s Death Round	The Last Node’s Death Round
0.1	875	2181
0.2	1051	1969
0.3	1064	1931
0.4	1066	1820
0.5	1055	1808
0.6	1035	1813
0.7	1036	1881
0.8	1037	1809
0.9	1038	1793

**Table 4:** A performance comparison between the four clustering protocols in the homogeneous networks (initial energy = 0.4 J).

Protocol	Stable Time	Unstable Time	Network Lifetime	Final Throughput
LEACH	654	275	929	5404
SEP	663	252	915	5357
DEEC	680	332	1012	7072
Our Protocol	767	507	1274	10,248

**Table 5:** A performance comparison between the four clustering protocols in the homogeneous networks (initial energy = 0.8 J).

Protocol	Stable Time	Unstable Time	Network Lifetime	Final Throughput
LEACH	1320	499	1819	10,099
SEP	1345	470	1815	10,336
DEEC	1326	718	2044	13,590
Our Protocol	1548	994	2542	20,432

**Table 6:** A performance summary between the four clustering protocols in the homogeneous networks.

Protocol	CHs Election Metric	Node Energy Distribution	Lifetime Ranking	Stable time Ranking	Throughput Ranking
LEACH	Random	Unbalanced	3rd	2nd	3rd
SEP	Initial energy	Unbalanced	3rd	2nd	3rd
DEEC	Initial energy Residual energy	Comparative balanced	2nd	2nd	2nd
Our Protocol	Residual energy Location of the node	Balanced	1st	1st	1st

**Table 7:** A performance comparison between the four clustering protocols in the homogeneous networks (the initial energy is evenly distributed at 0.4–0.8 J).

Protocol	Stable Time	Unstable Time	Network Lifetime	Final Throughput
LEACH	746	865	1611	8017
SEP	674	1018	1692	10,591
DEEC	813	1058	1871	12,426
Our Protocol	1064	867	1931	16,418

**Table 8:** A performance comparison between the four clustering protocols in the heterogeneous networks.

Protocol	CHs Election Metric	Node Energy Distribution	Lifetime Ranking	Stable time Ranking	Throughput Ranking
LEACH	Random	Unbalanced	4th	3rd	4th
SEP	Initial energy	Unbalanced	3rd	4th	3rd
DEEC	Initial energy Residual energy	Comparative balanced	2nd	2nd	2nd
Our Protocol	Residual energy Location of the node	Balanced	1st	1st	1st
